# Leveraging Community Engagement and Human-Centered Design to Develop Multilevel Implementation Strategies to Enhance Adoption of a Health Equity Intervention

**DOI:** 10.21203/rs.3.rs-5702080/v1

**Published:** 2025-03-28

**Authors:** Maggi A Price, Patrick J Mulkern, Madelaine Condon, Marina Rakhilin, Kara Johansen, Aaron R Lyon, Lisa Saldana, John Pachankis, Sue A Woodward, Kathryn M Roeder, Lyndsey R Moran, Beth A Jerskey

**Affiliations:** Boston College School of Social Work; Boston College School of Social Work; Boston College School of Social Work; Boston College School of Social Work; Boston College School of Social Work; University of Washington Department of Psychiatry and Behavioral Sciences; Chestnut Health Systems Inc; Yale University School of Public Health; Boston Child Study Center; Boston Child Study Center; Boston Child Study Center; Boston Child Study Center

**Keywords:** health equity, implementation strategies, implementation determinants, implementation intervention, multilevel, transgender, community-engaged research, human-centered design

## Abstract

**Background:**

Health equity intervention implementation (which promotes positive health outcomes for populations experiencing disproportionately worse health) is often impeded by health-equity-specific barriers like provider bias; few studies demonstrate how to overcome these barriers through implementation strategies. An urgent health equity problem in the U.S. is the mental health of transgender youth. To address this, we developed Gender-Affirming Psychotherapy (GAP), a health equity intervention comprising best-practice mental health care for transgender youth. This paper details the identification of implementation determinants and the development of targeted strategies to promote provider adoption of GAP.

**Methods:**

This study represents part of a larger study of mental health provider adoption of GAP. Here we describe the first 2 stages of the 3-stage community-engaged and human-centered design process – *Discover, Design/Build, and Test* – to identify implementation determinants of adoption and develop implementation strategies with transgender youth, their parents, and mental health providers. This process involved collecting data via focus groups, design meetings, usability testing, and champion meetings. Data were analyzed using rapid and conventional content analysis. Qualitative coding of implementation determinants was guided by the Health Equity Implementation Framework, and implementation strategy coding was facilitated by the ERIC Implementation Strategy Compilation.

**Results:**

We identified 15 determinants of GAP adoption, and all were specific to the transgender population (e.g., inclusive record system, anti-transgender attitudes). Seventeen implementation strategies were recommended and 12 were developed, collectively addressing all identified determinants. Most strategies were packaged into an online self-paced mental health provider training (implementation intervention) with 6 training tools. Additional inner setting strategies were designed to support training uptake (e.g., mandate training) and GAP adoption (e.g., change record system).

**Conclusions:**

Community-engaged and human-centered design methods can identify health equity intervention implementation determinants and develop targeted strategies. We highlight five generalizable takeaways for health equity implementation scientists: (1) implementer bias may be a key barrier, (2) experience with the health equity population may be an important facilitator, (3) stakeholder stories may be an effective training tool, (4) inner setting-level implementation strategies may be necessary, and (5) teaching implementers how to build implementation strategies can overcome resource-constraints.

**Trial registration:**

NCT05626231

## Introduction

Health equity promotion is an increasingly important goal for implementation scientists (1–5). Scholars recommend community-engaged research methods for identifying and overcoming barriers to health equity intervention implementation (1, 6–9). Specifically, best practices involve members of populations experiencing health inequities and implementers throughout the implementation process, in order to maximize reach and uptake. This community-engaged process involves identifying barriers, then developing, evaluating, and implementing strategies targeting those barriers. Despite the growing number of calls to action, very few implementation studies to date have employed these methods (8, 10–12).

### Health Equity Problem: Transgender Youth Mental

An urgent health equity problem in the U.S. concerns the disproportionate burden of adverse mental health borne by transgender youth (whose gender differs from their birth-assigned sex; 7). Compared to cisgender youth (whose gender aligns with their birth-assigned sex), transgender youth are 2–3 times more likely to be diagnosed with depression or anxiety (13, 14) and 6 times more likely to attempt suicide (15, 16). This inequity is exacerbated by the anti-transgender sociopolitical climate (7, 17–21) and exemplified by the uptick of state-level bans on evidence-based gender-affirming medical care for transgender youth (e.g., hormone treatment; 22–26). Gender-affirming medical care has been shown to be associated with improved mental health (27–38). Likewise, anti-transgender policies are linked to worse mental health and victimization among transgender youth (17, 39, 40). Access to effective mental health care is thus critical for transgender youth to combat these negative outcomes.

#### Affirming Mental Healthcare: Brief Overview of Effectiveness and Implementation Research

Affirming mental healthcare is designed to support a patient’s gender (and often sexual) identity(ies) and experiences and includes practices like using a patient’s affirmed (i.e., chosen) name and helping patients combat internalized bias through cognitive strategies (41–43). Several RCTs support the effectiveness of affirming mental health care, demonstrating that patients who receive affirming care experience significantly more treatment engagement and mental health improvements (e.g., more significant decreases in depressive symptoms) compared to those who do not (44–51). Importantly, however, relatively few trials of affirming care employ randomization due to the considerable ethical drawbacks of assigning vulnerable populations to care that is known to be less helpful (7, 52–55) and lacks acceptability for transgender patients (56–58). There are numerous non-randomized trials on affirming care, all of which demonstrate that it outperforms non-affirming care and waitlist conditions (e.g., steeper improvements in cognitive skills and depression; 57,59–61) on measures of treatment satisfaction, engagement, and mental health (e.g., depression, anxiety; 51,63,64). As an exhaustive review of effectiveness research on affirming mental healthcare is beyond the scope of this paper, we recommend a few excellent review papers: Burger and Pachankis (64), Tudor-Sfetea and Topcio (65), and Exposito-Campos (66).

The robust evidence on the effectiveness and acceptability of affirming mental health care has led to calls for relevant implementation studies (7, 67, 68), which scholars have recently begun responding to by evaluating fidelity (51, 69), feasibility (48, 57, 60, 70, 71) and novel implementation strategies (59, 72–78). These practices have also been codified into clinical treatment guidelines by national professional and accrediting bodies in mental health care, such as the American Psychological Association (64) and the American Psychiatric Association (38). Despite this empirical and clinical progress, transgender patients are often unable to access affirming mental healthcare because of provider bias communicated through treatment refusal or microaggressions (62, 80–83), and a dearth of providers trained in affirming mental health practices (84–86).

To address the widespread need for transgender-competent mental health providers, we developed Gender-Affirming Psychotherapy (GAP), an evidence-informed treatment tailoring approach (not a standalone intervention, which enhances scalability; 87) through a rigorous 4-year NIH-funded human-centered design intervention development project (88) involving: a scoping review of research literature (see Additional File 1 for scoping review references, previously published as a supplemental file; 43) and best practice guidelines (e.g. American Psychological Association), and human-centered design (HCD)-driven intervention refinement over one year via focus groups and interviews with community stakeholders (transgender youth, their parents, and providers; 43). GAP consists of evidence-informed and community-endorsed practice modifications encompassing 27 principles (knowledge that guides practice) and 38 skills (techniques or behaviors to use or avoid). The complete list of GAP principles and skills is available in Box 1 of Price et al. (43).

### Contributions to Gender-Affirming Practice Research and Health Equity Implementation Science

To facilitate the implementation of GAP, this study sought to systematically identify implementation determinants of GAP adoption and address them through the development of targeted implementation strategies. Very few studies targeting health equity problems identify determinants *and* design targeted implementation strategies (see exceptions led by Arnold 89, Cabassa 90–92, Oetzel 93,94, and Rogers 95). Specific to gender-affirming practices, no known previous research has identified mental health-care-specific determinants or strategies. Doing so is critical because mental health care is the only care setting where gender-affirming practices remain legal across the U.S. Nonetheless, studies have identified determinants of gender-affirming practice implementation in other settings (e.g., medical care, schools) spanning multiple levels; example determinants include implementer knowledge, implementer attitudes, institutional climate, and workload. (7, 96–99)

Research on implementation strategies for gender-affirming practice adoption is also scarce, though some have recommended strategies (e.g., medical training, appeal insurance denials) based on their clinical experience and literature synthesis (100, 101). Evaluations of in-person training suggest that it can enhance implementer knowledge about gender-affirming practices and improve attitudes toward transgender people (78, 102). While promising, these trainings have not typically been systematically developed and tested, or they focused on affirming practices broadly (including sexual minority affirming practices). This study contributes to the growing literature on gender-affirming practice adoption specifically, and more broadly, to the use of best practices in health equity implementation science (1–5, 10, 103, 104) to identify and develop multilevel implementation strategies to target a major health equity problem.

### Current Study

We employed community-engaged human-centered design (HCD) methods to identify implementation determinants (barriers and facilitators) and implementation strategies (methods to promote an implementation outcome) to support GAP adoption among mental health providers. Implementation strategies were then designed and refined to address the identified determinants. This paper details a replicable process for systematically identifying and addressing health equity intervention determinants in collaboration with affected communities and describes the resulting implementation strategies.

To ensure a comprehensive assessment of health equity factors, we utilized the Health Equity Implementation Framework (HEIF; 4) - a determinants framework tailored to identify multilevel barriers and facilitators of health equity intervention implementation - throughout the research process. We chose the HEIF over other determinant frameworks because it determines whether an implementation determinant is specific to a health inequity and highlights determinants at the structural level, which are often central to health equity, but regularly overlooked. In this study, we use the HEIF to identify determinants (its original purpose) *and* to inform implementation strategy development alongside the Expert Recommendations for Implementing Change (ERIC) Compilation of Implementation Strategies (105–108).

## Methods

### Overview and Procedural Framework

**Figure 1.**
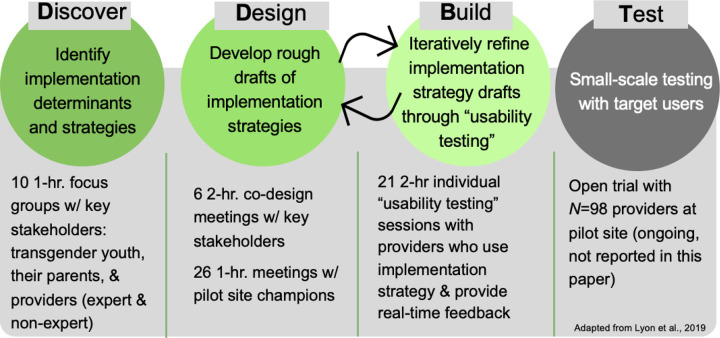
Process for Identifying Implementation Determinants and Developing Implementation Strategies

This study represents part of a larger project evaluating implementation strategies to promote mental health provider adoption of GAP (109). To identify implementation determinants of GAP adoption and develop implementation strategies targeting those determinants, we completed the first 2 of 3 stages of *Discover, Design/Build, and Test (DDBT),* an community-engaged HCD framework for developing and refining interventions and implementation strategies (see framework and procedures in Fig. 1; 110,111). Across stages, we worked closely with community stakeholders central to the implementation goal (GAP adoption): transgender youth, their parents, and mental health providers, including those with and without expertise working with transgender youth. In the *Discover* stage, we identified implementation determinants and strategies, and in the *Design/Build* stage, we developed the previously discovered implementation strategies. The *Test* stage will be described in a forthcoming paper.

The larger study involved a research-practice partnership with a multi-site mental health agency headquartered in the Northeast serving youth on the East and West Coasts. We chose this agency because it is a setting that does not specialize in care for LGBTQ youth but has a growing transgender patient population. To enhance the long-term reach of GAP (112), it was important to evaluate a setting representative of mental health care services for the target population, but not one where providers are expected to already have GAP competency (113). Researchers met virtually with partner-agency provider “champions” (who promote and facilitate the implementation of an innovation) throughout the duration of the study. Champions were 4 partner-agency leaders (i.e., held director positions) with varying GAP expertise who supported GAP implementation. The champions represented a previously established group of providers invested in research-partnerships who met weekly about evidence-based practices (e.g., measurement-based care). We analyzed champion meeting data for this study because it informed implementation determinant identification and strategy development. Reporting for this study follows the Standards for Reporting Qualitative Research (114) (SRQR; see Additional File 2).

### Recruitment and Enrollment

Participant recruitment involved purposive sampling (115) and social media (116, 117). We sought to recruit a sample that was racially representative of Boston (participants’ primary location) and achieved this goal. The [redacted] IRB approved the study. All participants resided in the Northeast U.S. and provided informed consent. Table 1 provides participant demographics and provider professions. Additional details about our sample and procedures are in [redacted citation].

### Procedures, Sample, and Analyses

#### Discover Stage

*Discover* focused on identifying implementation determinants and strategies through 10 separate virtual focus groups (February-April 2022) with transgender youth (*n* = 6, ages 13–23; 3 meetings), parents of transgender youth (*n* = 3; 1 meeting), mental health providers with expertise working with transgender youth (*n* = 11 providing 2 + years gender-affirming care; 3 meetings), and without expertise (*n* = 7; 3 meetings). Semi-structured interview protocols were used (full protocol in Additional file 3).

#### Design/Build Stage

*Design/Build* focused on developing implementation strategies, first by drafting previously identified implementation strategies (*Design*) and then refining those drafts (*Build*)*. Design* involved six 2-hour virtual meetings (June-July 2022) with separate groups of community stakeholders (4 youth; 2 parents; 12 providers). Meetings involved participants, professional designers, and researchers collaborating on *MURAL* (118), an online tool for visual collaboration on a digital canvas in real-time through idea sharing (e.g., sticky notes, images) and information organizing. Meeting interview protocols and canvases are in Additional file 4. Throughout this stage, meeting notes and video recordings were reviewed and rapidly analyzed (119) by the PI (first author) and project coordinator/co-builder (fourth author) using the HEIF to facilitate the generation and iterative refinement of a list of suggested implementation strategies. Those that could be feasibly built within study constraints (time, budget, scope) were drafted. For example, the strategy “develop educational materials” involved researchers drafting and editing curriculum on google docs. Once edited, the educational materials were transferred to an online Learning Management System, the primary platform used to facilitate *Build*.

*Build* involved refining implementation strategy drafts through “usability testing.” Specifically, 21 2-hour individual usability testing sessions (November 2022-January 2023) were conducted with providers representing target users (*n* = 4). Sessions involved a participant interacting with implementation strategies (e.g., training materials) while being observed by a researcher, and providing real-time feedback on usability and acceptability. Participants were directed to “think aloud,” meaning vocalizing thoughts and feelings while engaging with material; researchers prompted participants with open-ended questions to encourage elaboration as needed (e.g., “Why did you answer the way you did”; 120). The usability testing protocol is in Additional File 5. Researchers addressed usability issues throughout this stage, ensuring that implementation strategies were ready for subsequent testing.

#### Champion Meetings

Per the request of the partner agency, weekly meetings between GAP champions and researchers were initiated in July 2022 (still ongoing). These meetings informed the prioritization and tailoring of implementation strategies for the partner agency. Accordingly, we analyzed detailed notes from the 36 weekly 30- to 60-minute meetings held from the initiation of the partnership through the start of the *Test* stage (July 2022-May 2023). Champion meetings coincided with their pre-existing meeting (i.e., a portion of the pre-existing meeting was dedicated to GAP) and focused on agency-specific GAP adoption. All champions consented to participate in the subsequent test phase of the larger project; demographic information is not provided herein to protect their confidentiality.

#### Conventional Content Analysis

Building on rapid qualitative analyses conducted throughout data collection, we re-analyzed the data using conventional content analysis (121) 3; February-August 2024) to validate and synthesize results. Data included transcripts from the *Discover* and *Design/Build* stages, and comprehensive meeting notes from champion meetings. Data were coded by the second and third authors, who met with the first author weekly to build consensus through reviewing codes and resolving discrepancies (122). During meetings, researchers identified and reflected on how their identities, experiences and biases may have influenced their interpretation of the data (123, 124). Of note, our research team represents diverse gender identities (e.g., transgender, nonbinary, cisgender) and we are all proponents of GAP. We hold varying levels of GAP expertise, and many of us have ample clinical experience treating transgender youth.

Implementation determinants were coded deductively (guided by a codebook developed during rapid qualitative analysis) and inductively (allowing new codes to emerge). Implementation determinants were categorized using the HEIF, by both determinant level (e.g., outer setting) and whether or not the determinants were health equity related. Health equity determinants are those specific to the health equity population (transgender youth) and uniquely influence implementation (provider adoption of GAP). Implementation determinants unrelated to health equity are common across populations and interventions (e.g., funding). Determinant code frequencies were calculated to inform the synthesis of results. Implementation strategies were deductively coded, such that each code represented a discrete implementation strategy in the Refined ERIC Compilation (106–108). Implementation strategies were categorized using both Waltz’s implementation strategy categories (e.g., train/educate, involve consumers; 106) and HEIF levels (e.g., recipient-level, like patients and providers, inner setting-level, like clinic; 4,105).

## Results

### Determinants

**Figure 2.**
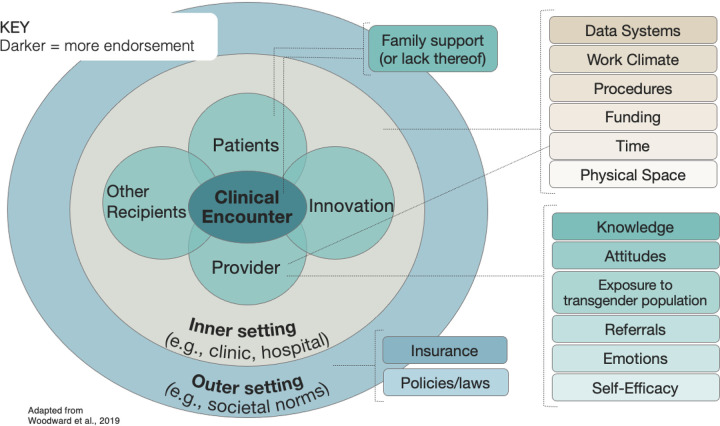
Determinants of Gender-Affirming Psychotherapy Adoption Organized within the Health Equity Implementation Framework

*Discover* data analysis revealed 15 determinants of GAP adoption across all HEIF levels; 13 were categorized at one level and 2 at two levels. Among single-category determinants, 6 were at the provider level, 5 were at the inner setting-level, and 2 were at the outer setting-level. The two double-classified determinants were: 1) “family support” for the youth’s gender, categorized at both patient and clinical encounter-levels because it reflected a patient factor (e.g., youth not disclosing their gender identity to their parents) and affected the clinical encounter (e.g., in parent sessions); and 2) “time,” categorized as both provider and inner setting-levels because it referred to time under the control of the provider (e.g., limited time for training due to a large private practice caseload) or organization (e.g., no organizationally protected time for training). Determinants and their levels are shown in Fig. 2. All but one determinant was identified by participants as both a barrier and facilitator; “policy” (political, organizational, and professional rules and regulations affecting GAP adoption) was the exception, solely discussed as a barrier. Twelve of 15 determinants were primarily endorsed as barriers (i.e., more often discussed as barriers than facilitators; details in Table 2). The 4 most commonly endorsed determinants were: provider knowledge (about gender-affirming practices and/or transgender youth; coded 83 times), provider attitudes (positive or negative towards transgender youth and/or gender-affirming practices; coded 53 times), family support (for the youth’s gender; coded 44 times), and provider exposure (to transgender youth, including in professional and personal settings; coded 23 times).

### Health Equity Focus

HEIF-guided content analysis revealed that all 15 implementation determinants were specific to the health equity population (transgender youth) with 4 *also* reflecting implementation determinants unrelated to the population (see * in Table 2). For example, regarding the determinant self-efficacy, participants expressed concerns that were both general (“I haven’t practiced this enough and I really want to get better at it”) and population-specific (“I’m intimidated to take someone [transgender] on because I just don’t feel like I’m equipped yet to do that.”; “What if they prefer I use certain pronouns with different people and I mess up?”). Additional file 6 provides exemplar quotes.

### Implementation Strategies

**Figure 3.**
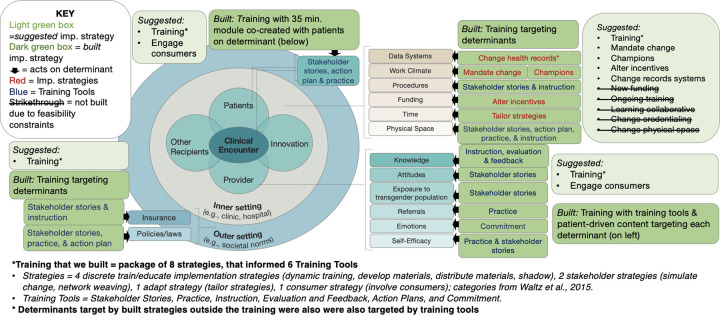
Implementation Strategies

Seventeen of 73 discrete ERIC implementation strategies (107) across 6 of 9 of Waltz’s implementation strategy categories (106) were suggested; of these, 12 strategies across all 6 categories were built. Importantly, the strategies collectively addressed every previously identified determinant. Built implementation strategies are detailed below, summarized in Table 3, and examples are in Additional file 7. The five strategies that were not built (e.g., learning collaborative) are listed in Fig. 3; see Additional file 8 for descriptions and rationale for not building each (e.g., funding).

### Standalone Implementation Intervention: Training Incorporating Eight Discrete Strategies and Six Training Tools

Eight discrete strategies (detailed parenthetically upon first mention) were packaged within an 8-hour training tailored to address 12 of the 15 determinants. The training - primarily designed to impart information and build skills - can be classified as an implementation intervention because it represents a bundled set of strategies (125), but we use the term “training” herein for clarity. The training was designed with community stakeholders (strategy 1: develop materials). It was tailored to meet the work-related needs of provider participants (strategy 2: tailor strategies) such that it was online, self-paced, comprehensive (for beginners and expert clinicians), delivered through text and read-aloud AI-driven-technology, and offered over 2 months during a slow work period determined in advance by the partner agency (strategy 3: distribute materials). The training consisted of 10 modules (i.e., self-contained training segments focused on specific topics), each of which aligned with the 10 domains of the GAP clinical intervention (109) and involved training tools recommended by stakeholders (strategy 4: dynamic training) across 6 categories: stakeholder stories, practice, instruction, evaluation and feedback, action plans, and commitments.

#### Training Tool 1: Stakeholder Stories.

**Figure 4.**
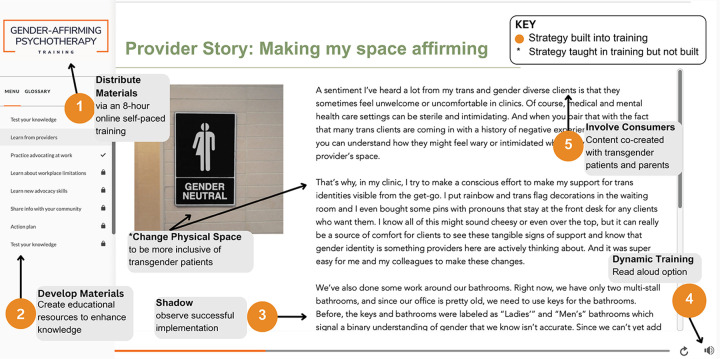
Example of Implementation Strategies Built in Online Training

The training incorporated 43 stories from the anonymized perspectives of youth, parents, and providers (stakeholders), co-written by researchers and community stakeholders (strategy 5: involve consumers) based on data collected in the *Discover* and *Design* stages. To enhance exposure to the population, all stories had a read-aloud option with a voice matching the affirmed gender of the stakeholder, and a stock photo representing them. Several stories also demonstrated how mental health providers implemented GAP in real-world clinical settings (strategy 6: shadow). See Fig. 4 for an example of how several discrete implementation strategies were combined to create and deliver a stakeholder story.

#### Training Tool 2: Practice.

Eighteen practice activities required rehearsal of a key skill. For example, after learning about *gender dysphoria* (i.e., distress some transgender youth experience when their birth-assigned sex differs from their gender), providers read and/or listened to an example session of a provider and patient discussing the drawbacks and benefits of a gender dysphoria diagnosis (strategy 6: shadow). Next, providers practiced; they were given the prompt “explain what gender dysphoria is and why (or why not) you might diagnose a client with gender dysphoria” and practiced using their own words to write or audio-record a response (strategy 7: simulate change). Another practice activity involved providers identifying 2–3 local gender-affirming medical providers; their responses were added to a shared Google spreadsheet serving as a referral list that could be accessed during and after the training (strategy 8: network weaving).

#### Training Tool 3: Instruction.

Thirty-five instances of instruction presented foundational concepts and actionable, step-by-step skill guidance in accessible language. Complex topics, like anti-transgender legislation, were taught in multiple ways. For example, providers first received information about the recent rise in anti-transgender legislation (20). Next, providers were given information about the relevance of these policies to their work, such as the potential mental health effects this legislation may have on their transgender patients (e.g., increased suicidal ideation; 19). Finally, they were taught how to find their state’s policies affecting transgender youth (126), which was reinforced with an opportunity to practice discussing the legislation with a hypothetical patient.

#### Training Tool 4: Evaluation and Feedback.

Twenty evaluations, consisting of 5-item quizzes administered before and after each training module, assessed module-specific knowledge acquisition. Each pre-module quiz was scored immediately; if any item was incorrect, providers were shown “You are still learning and that’s ok! Let’s continue through the course.” After each post-module quiz, correct answers and associated explanations were provided to reinforce knowledge acquisition (127).

#### Training Tool 5: Action Plans.

Providers created 6 action plans documenting their goals and intentions to adopt GAP skills. Each action plan could be downloaded and trainees were encouraged to save them for future accountability. Action plans were presented after stakeholder stories or instruction as an opportunity for providers to apply what they learned. For example, after four stakeholder stories about providers succeeding or failing to use GAP, providers were given the action plan prompt: “Please share three practices that you will use with caregivers of transgender youth that you learned from the stories in this module.” The final action plan provided recommended activities to support GAP adoption beyond the training, many of which were implementation strategies suggested in the present study but not built due to feasibility constraints (e.g., start a learning collaborative, advocate for policy reform).

#### Training Tool 6: Commitment.

Commitments are a behavioral change technique believed to facilitate behavior change (127, 128) by eliciting an active commitment to a specified behavior (129). Providers were asked to affirm their commitment to learning GAP at the beginning of each of the 10 modules. For example, at the start of a 25-minute module providers were asked, “Do you commit to completing the next 25-minute module?” and clicked either “yes” or “no.”

### Training Content to Address Inner and Outer Setting Determinants

Within the training we addressed 4 inner setting determinants (data systems, work climate, procedures, physical space). For example, we targeted “data systems” through 4 types of content (each representing a different “training tool,” described above): 1) instruction on being transparent with patients about the limitations of the record systems (e.g., limited gender options, presence of deadname), 2) a provider and client story detailing the benefits of affirming record systems and advocating to change a record system, respectively, 3) an action plan wherein providers commit to advocating for a record system with inclusive name, pronoun, and gender identity fields, and 4) an evaluation and feedback opportunity assessing providers’ knowledge about how to discuss record system limitations with patients.

Similar training content targeted 2 outer setting determinants (insurance, policies/laws). For instance, the training taught implementers how to change physical space through 1) a provider story chronicling their advocacy to relabel the clinic bathrooms, 2) instruction on why inclusive bathrooms are important, 3) practice discussing the importance of inclusive bathrooms with a coworker, and 4) an action plan wherein providers commit to advocating for gender-inclusive spaces in their own workplace.

### Four Built Inner Setting Implementation Strategies

Our strong research-practice collaboration with the partner agency allowed us to build four implementation strategies targeting three inner setting implementation determinants (data systems, work climate, and funding) typically constrained or prevented by real-world feasibility factors like money and organizational buy-in. These four strategies included modifying the electronic health record system to enhance gender-inclusivity (e.g., affirmed name, pronouns; strategy 9: change record system), the partner-agency CEO requiring providers to complete the training (but not the study; strategy 10: mandate change), establishing a group of champions who met regularly to promote organization-wide GAP training and adoption (strategy 11: champions), and providing continuing education credits (CEs) to training completers (strategy 12: alter incentives). To help ensure that the CE incentive strategy was feasible long term, researchers directly applied for national CE accreditation for psychologists, mental health counselors, and social workers.

## Discussion

Using community-engaged human-centered design methods, we collaborated with transgender youth, their parents, and mental health providers to identify implementation determinants and develop implementation strategies to promote mental health provider adoption of a health equity intervention (Gender-Affirming Psychotherapy). Results revealed 15 determinants of GAP adoption across all levels of the HEIF. Of the 17 suggested implementation strategies, 12 were identified as feasible and developed, collectively addressing all determinants. Notably, 8 of the strategies were packaged within an implementation intervention; specifically an innovative online training with 6 training tools. In this discussion, we synthesize our findings across 5 key takeaways in the hopes of guiding future health equity intervention researchers and implementers.

### Takeaway 1: Implementer Bias May Impede Health Equity Intervention Implementation

Provider attitudes, like anti-transgender bias, were the second most commonly endorsed determinant of GAP adoption, after knowledge. Implementation research on attitudes focuses almost exclusively on attitudes about using a particular practice (129), not on attitudes toward the patient population. While several commentaries about health equity-focused implementation science have encouraged researchers to evaluate provider biases (10, 105, 130), few studies have (4, 95, 131). Nonetheless, it is well-established that providers’ biases about health equity populations (e.g., implicit and explicit racism) negatively affect patient engagement and healthcare outcomes (132–134). It is thus unsurprising that such biases were identified by stakeholders (transgender youth, their parents, and providers) as barriers to using practices that support health equity populations, such as those in GAP. Echoing other health equity implementation researchers, we argue that measuring implementer attitudes about the patient population (vs. only the practices) is critical. The present study offers data to support this argument and provides concrete implementation strategies to address implementer bias.

### Takeaway 2: Experience With the Health Equity Population Can Facilitate Health Equity Intervention Implementation

Provider bias in health care is attributable to factors like poor skills in culturally-responsive care, lack of knowledge about the patient population, and lack of experience with the patient population (135, 136). Mirroring these findings, our participants shared that intervention adoption requires more than just intervention skills; it requires knowledge *about* the health equity population and experience *with* the population. Given the online and self-paced nature of our training, we were unable to utilize some common strategies used in medical provider education, like practice with standardized patients (137) and patient-teacher-led presentations (138). Instead, we used multimodal and exposure-based training tools endorsed by our participants that could be built into a self-paced training. Importantly, training tools involving rehearsal have been shown to be effective in other behavioral interventions (139, 140). An example in our study is practice activities, which require providers to write or audio-record hypothetical responses to a patient after reading and/or listening to dialogue between a patient and provider. Another example is stakeholder stories, which we expand on directly below.

### Takeaway 3: Stakeholder Stories May Address Barriers Like Implementer Bias And Emotion, and Leverage Facilitators Like Exposure to The Health Equity Population

Consistent with extant literature, we believe that stakeholder stories are a potentially powerful training tool (141–143), and encourage health equity intervention researchers to co-create them with stakeholders and include them in their implementation efforts. We suspect that patient stories in particular may help reduce bias based on ample evidence supporting contact theory, the social science theory positing that intergroup contact can reduce prejudice (144–148). Our training included substantial stakeholder stories - specifically, narratives from the perspectives of patients (transgender youth), implementers (mental health providers), and other recipients (parents of transgender youth).

Drawing on the Information Motivation Behavior Model o*f behavior change* (149–151) - which posits that behavior change results from enhanced knowledge, self-efficacy, and attitudes (also identified as top determinants in this study) - all stories sought to enhance knowledge (i.e., included key facts about the population and/or GAP skills) and several provided an opportunity to “shadow” provider behavior change to enhance self-efficacy. To improve attitudes towards the population and/or GAP practices (e.g., acceptability, appropriateness), patient stories were designed to elicit empathy (e.g., about patients’ lived experiences) and demonstrated how GAP practices benefited patients. In addition to targeting several determinants simultaneously, stories leveraged multiple discrete implementation strategies (e.g., involve consumers, simulate change), and often did so in a single story (example in Fig. 4). In sum, stakeholder stories may be an especially efficient and effective training tool that future studies should evaluate.

### Takeaway 4: Inner Setting Implementation Strategies May Be Necessary for Health Equity Implementation

Implementation researchers consistently highlight the necessity of inner setting-level (e.g., clinic, hospital) implementation strategies (152). In this study, at least one organizational strategy was necessary for comprehensive GAP adoption: changing record systems, which involved modifying the partner agency’s electronic health record system to enhance the inclusivity of patients’ name and gender options (details in Table 3). This strategy was necessary because GAP practices include asking and recording a patient’s affirmed name, pronouns, and gender (i.e., aligning with one’s true identity 43); in other words, if there was no way to record these data, providers could not fully adopt GAP. While the other implementation strategies (mandate change, champions, alter incentives) may not be absolutely necessary for GAP adoption, each targeted inner setting determinants, namely work climate and funding. Echoing other health equity implementation researchers, health equity intervention implementation may be especially dependent on implementation strategies targeting inner setting determinants like workplace climate (e.g., the extent to which an organization supports equity and justice efforts) and funding to support new programs (e.g., bias training 10,105).

### Takeaway 5: Teaching Implementers How to Build Implementation Strategies Can Overcome Resource-Constraints

Though inner setting implementation strategies may be important, they are often costly. The identified implementation strategies that we did *not* build due to financial and personnel constraints targeted determinants at the inner setting (e.g., change physical space). In addition, three of the four built inner-level strategies were limited in scope, such that they could only benefit providers working for our partner agency (the exception was CEs). To maximize the potential scalability of built inner setting implementation strategies, we included content in our training on how to build these implementation strategies as an implementer. Teaching implementers how to build and/or advocate for inner setting-level implementation strategies is likely a cost-effective and scalable alternative to building implementation strategies.

### Strengths and Limitations

Our study has several important strengths. It illustrates the process of identifying implementation determinants and building targeted implementation strategies to address a major health equity problem. Many commentaries make recommendations for health equity implementation research (2, 8, 10), and some studies have either identified health equity determinants (4, 95, 131) or adapted implementation strategies to address health equity (153–156); but we are aware of few that achieve both (see exceptions (89–92). Second, we employed many of the recommended best practices for conducting health equity implementation research (10, 112). For example, we used community-engaged research methods and HCD methods that center the needs of the health equity population, chose a patient-endorsed intervention, and addressed contextual determinants. Third, we contribute what we believe is the first study to apply the HEIF (a determinant framework) to implementation strategy identification and building (representing the “facilitation” portion of the HEIF; 4,105). Finally, we believe our findings (e.g., determinants, takeaways), including the extensive detail we provide on procedural methods and results (see Additional Files), have the potential to be a generalizable resource for other health equity implementation researchers invested in utilizing best practices.

Alongside these strengths are a few key limitations. First, we did not collect prioritization and/or feasibility data on implementation determinants and strategies (e.g., using validated surveys like the pragmatic context assessment tool (157) and the inventory of factors affecting successful implementation and sustainment (158)) or conduct implementation mapping (159). Instead, we used qualitative analysis to rank-order determinants and strategies based on level of endorsement (i.e., how many times they were coded) and iterative implementation strategy development (i.e., HCD methods) to prioritize, build, and refine implementation strategies. While our approach has its merits (e.g., engaging stakeholder collaboration), collecting survey data on prioritization and feasibility would add clarity to our findings and may have resulted in different built implementation strategies. Second, we built all implementation strategies that were feasible. While doing so may be appropriate for the development phase, the result was a combination of many implementation strategies: 8 embedded within one implementation intervention and 4 separate inner setting strategies. While we are currently in the process of testing these strategies, given their bundled and complex nature, we will be unable to assess which are most potent in the initial evaluation. Nonetheless, the comprehensive and longitudinal data we are collecting will enable us to evaluate the mechanisms (namely, knowledge, attitudes, and self-efficacy) through which the implementation intervention may operate. As noted by leading implementation scholars (160), we may have avoided building so many strategies if we had used alternative approaches, like the CFIR-ERIC Implementation Strategy Matching Tool (108, 161). Finally, our sample was primarily located in the Northeast, a region with relatively low anti-transgender bias (17). Accordingly, identified determinants and strategies may not be generalizable to U.S. regions with more anti-transgender bias, potentially limiting the reach of GAP.

## Conclusion

This paper details the rigorous use of best practices in health equity implementation science (e.g., community-engaged methods; 1–5,10,103,104) to develop targeted multilevel implementation strategies to address a major health equity problem. Importantly, we also used these methods to develop the intervention (43), engaging the community across all early stages of intervention development and implementation (see exceptions led by Cabassa 90–92, and Oetzel 93,94). This study suggests that community-engaged and HCD methods can be successfully utilized to identify determinants and develop targeted multilevel implementation strategies across all HEIF levels to facilitate the implementation of a health equity intervention. In an effort to support other health equity researchers conducting implementation studies, we provide ample detail about the study process and results (see also Additional files). Finally we provide five generalizable takeaways for researchers and implementers invested in promoting the adoption of health equity interventions: (1) implementer bias may be a key barrier, (2) experience with the health equity population may be an important facilitator, (3) stakeholder stories may be an effective training tool, (4) inner setting implementation strategies may be needed, and (5) teaching implementers how to build implementation strategies can overcome resource-constraints.

## Figures and Tables

**Table 1:** Youth, Parent, and Provider Demographics Across Discover and Design/Build Stages

	“Discover” Stage				“Design/Build” Stage				Total (across stages)		
	Focus Groups				Design Meetings			Usability Testing			
	Youth *(n = 6)*	Parents *(n = 3)*	Expert Providers *(n = 11)*	Non-Expert Providers *(n = 7)*	Youth*** (n = 4)	Parents*** (n = 2)	Providers *(n = 12)*	Providers *(n = 4)*	Youth* (N = 8)	Parents* (N = 4)	Provide** (N = 25)
**Age Range (M)**	18–23 (22)	49–52 (51)	27–40 (34)	26–58 (36)	13–23 (18)	48–52 (50)	23–58 (34)	32–58 (47)	13–23 (20)	48–52 (50)	27–58 (36)
**Gender (n)**											
Cisgender man	0	0	2	0	0	1	1	0	0	1	2
Cisgender woman	0	3	8	5	0	1	8	3	0	3	18
Transgender man	2	0	0	0	4	0	0	0	4	0	0
Transgender woman	0	0	0	0	0	0	0	0	0	0	0
Nonbinary/genderqueer	3	0	1	1	0	0	2	1	3	0	4
Transgender and Nonbinary	1	0	0	0	0	0	0	0	1	0	0
Gender non-confirming woman	0	0	0	1	0	0	1	0	0	0	1
Race**** **(n)**											
Asian	2	0	1	1	0	0	0	0	2	0	2
Black/African American	2	0	1	0	1	0	2	1	2	0	3
White	3	3	8	6	2	2	9	3	3	4	18
Hispanic/Latino	0	0	1	0	1	0	0	0	1	0	1
Portuguese	0	0	0	0	0	0	1	0	0	0	1
**Profession (n)**											
Psychologist	-	-	0	0	-	-	0	1	-	-	1
Clinician	-	-	0	1	-	-	1	0	-	-	2
Social Worker	-	-	2	5	-	-	6	2	-	-	8
Counselor	-	-	2	1	-	-	0	0	-	-	3
Psychotherapist	-	-	0	2	-	-	1	0	-	-	3
Family & Child Services	-	-	1	0	-	-	1	0	-	-	1
Student/Trainee	-	-	0	0	-	-	1	0	-	-	1
Educator	-	-	1	0	-	-	1	0	-	-	2
Agency Administrator	-	-	2	0	-	-	1	0	-	-	3
Case Manager	-	-	1	0	-	-	0	0	-	-	1
**Practice Setting (n)**											
Group	-	-	5	1	-	-	4	2	-	-	9
Independent	-	-	5	4	-	-	4	2	-	-	11
School-Based	-	-	1	1	-	-	2	0	-	-	3
Community	-	-	0	1	-	-	1	0	-	-	1
Hospital	-	-	0	0	-	-	1	0	-	-	1

* 2 transgender youth participated in both Discover Focus Groups and Design Meetings; 1 parent participated in both Discover Focus Groups and Design Meetings.

** 1 provider participated in Discover Focus Groups, Design Meetings, and Build Usability Testing; 1 provider participated in Champion meetings and Build Usability Testing; 7 providers participated in both Discover Focus Groups and Design Meetings

*** The Design meetings featured one parent-child pair who separately participated in the parent and youth groups.

**** Respondents were able to self-identify with one or more races based on 2020 Census standards. One participant in the transgender youth Focus Group identified as both Black/African American and White. Purposive sampling was used during recruitment to recruit a racially representative sample of transgender youth, parents, and providers based on Boston-area census data.

**Table 2:** Determinant Descriptions, Endorsement Frequency, and % Endorsement by Participant Type

Determinants ordered from most to least frequently endorsed			Endorsement Frequency (total)			% Participants who Endorsed Determinant		
Determinant	HEIF Level (4)	Determinant Description	*Barrier* (95)	*Facilitator* (110)	*Sum* *(205)*	*Youth*	*Parent*	*Provider*
Knowledge	Provider	Provider’s comprehension, understanding, and awareness of transgender youth, and/or GAP. **Example:** Reviewing transgender-related vocabulary.	39	44	83	75%	25%	56%
Attitudes	Provider	Provider’s beliefs and/or values related to transgender youth and/or GAP. **Example:** Believing being transgender is a trend.	28	25	53	63%	25%	64%
Family	Patient	Youth’s family’s behaviors, characteristics, or values related to the youth’s transgender identity. **Example:** Parents refusing to use the youth’s affirmed name and pronouns.	41	3	44	63%	50%	44%
	Clinical Encounter							
Exposure to Transgender Population	Provider	Provider’s direct or indirect experiences with transgender youth. **Example:** Never having met a transgender person.	3	20	23	50%	0%	20%
Data Systems	Inner Setting	Structures for collecting, organizing, and sharing youths’ gender-related information. **Example:** Intake form only provides M and F as options for “gender.”	8	8	16	0%	0%	32%
Referrals	Provider	Connections made to other gender-affirming services. **Example:** A list of gender-affirming endocrinologists.	4	8	12	13%	75%	12%
Work Climate	Inner Setting	Organizational environment affecting GAP adoption. **Example:** Coworkers refusing to use a youth’s affirmed name.	7	4	11	0%	0%	28%
Emotions*	Provider	Provider’s feelings about GAP that affect use. **Example:** Fear of mistakenly using the wrong pronoun.	9	1	10	13%	0%	20%
Procedures	Inner Setting	Protocols and policies of an organization that influence adoption of GAP. **Example:** Intake form asking for dead/legal name.	7	2	9	0%	0%	20%
Insurance	Outer Setting	Insurance coverage policies that affect the use of GAP **Example:** Insurance documents requiring a youths’ dead/legal name.	8	1	9	25%	50%	4%
Self-Efficacy*	Provider	Provider’s self-perceived capability of using GAP that influences adoption. **Example:** Not feeling competent and wanting more practice before treating a transgender youth.	4	4	8	13%	0%	12%
Funding*	Inner Setting	Availability and allocation of personal and organizational resources to invest in facilitating and incentivizing GAP adoption. **Example:** Clinic does not offer funds to pay for GAP training.	6	2	8	0%	50%	16%
Time*	Provider	Availability of personal and organizational investment in GAP adoption. **Example:** Paid time for GAP training.	4	1	5	0%	0%	12%
	Inner Setting							
Policies	Outer Setting	Political, organizational, and professional regulations that affect transgender youth and GAP adoption. **Example:** Policy requiring transgender youth to get a gender dysphoria diagnosis from a mental health provider to access gender-affirming medical care.	5	0	5	25%	0%	8%
Physical Space	Inner Setting	Gender-inclusive spaces. **Example:** Bathrooms designated and labeled as “All Gender.”	2	1	3	0%	0%	8%

* Implementation determinants that *also* reflect determinants unrelated to the transgender youth population.

**Table 3:** Built Implementation Strategies Categories and Descriptions*

#**	Strategy(69)	Description	Category(68)
1	Develop educational materials	Developed educational content with community stakeholders (detailed in Results, “Standalone Implementation Intervention: Training Incorporating Eight Discrete Strategies and Six Training Tools”) to address all GAP clinical intervention skills and principles.	Educate/Train
2	Tailor strategies	Strategies were tailored to meet the needs of mental health providers, based on feedback from provider participants and champions. For example, the training is online and self-paced because providers said that their schedules could not accommodate an in-person training that required several consecutive hours.	Adapt and tailor to context
3	Distribute educational materials	The research team provided and oversaw the administration of (e.g., provided ongoing tech support) the online training. Throughout the training, providers had the option of downloading and/or saving several training materials (e.g., module summaries). After training completion, a PDF of the training was distributed to training completers.	Educate/Train
4	Dynamic Training	Training incorporated 6 teaching tools. Additional details and examples are described in the Results. Examples of training content are also provided in Fig. 4.	Educate/Train
5	Involve Consumers	The research team developed GAP and GAP implementation strategies in close collaboration with transgender youth and their parents. Stakeholder stories illustrate this strategy best (detailed further in Results “Stakeholder Stories”)	Engage consumers
6	Shadow	Training included provider stories and example sessions demonstrating GAP skills.	Educate/Train
7	Simulate change	Training included practice activities involving the simulated use of GAP practices. For example, providers practiced explaining gender dysphoria in their own words (detailed in Results “Training Tool 2: Practice”).	Develop stakeholder interrelationships
8	Network Weaving	Training included two practice activities focused on network weaving. In the first, providers joined a previously established referral list for gender-affirming care providers in the Northeast. The second activity is described in Results under Training Tool 2: Practice.	Develop stakeholder interrelationships
9	Change record systems	The partner clinic updated electronic health records (EHR) system (detailed further in Results, “Four Inner setting-level Implementation Strategies”)	Change Infrastructure
10	Mandate change	At 2 all-staff meetings (one before and one during the training period), the partner-agency CEO verbally emphasized the importance of GAP, noting that it aligned with the agency values and would be included in future conversations about promotions, raises, and funding for other trainings.	Change Infrastructure
11	Champions	The research team collaborated with partner agency leadership to establish a group of Champions (described in Methods, “Champion Meetings”)	Develop stakeholder interrelationships
12	Alter incentives	Researchers were accredited to provide continuing education units (CEs) through the National Association of Social Workers, American Psychological Association, and National Board of Certified Counselors. CEs were offered to incentivize training completion.	Finance

* Examples of built strategies are in Additional file 5; Suggested strategies that were not built are described in Additional file 6.

** Corresponding with order strategy was presented in-text

## Data Availability

The datasets generated and/or analyzed during the current study are not publicly available due to the sensitive nature of participant data

## Abbreviations

AI: Artificial Intelligence
CE: Continuing Education
CEO: Chief Executive Officer
CFIR: Consolidated Framework for Implementation Research
DDBT: Discover, Design/Build, Test Framework
ERIC: Expert Recommendations for Implementing Change
GAP: Gender-Affirming Psychotherapy
HCD: Human-Centered Design
HEIF: Health Equity Implementation Framework
